# Analysis of steroid hormones in shell eggs from layer breeds common to Taiwan by liquid chromatography–tandem mass spectrometry

**DOI:** 10.1002/fsn3.1074

**Published:** 2019-05-28

**Authors:** I‐Chen Li, Wen‐Yuan Yang, Chung‐Hsi Chou, Yi‐Chen Chen, Su‐Lien Kuo, Sheng‐Yao Wang

**Affiliations:** ^1^ Zoonoses Research Center and School of Veterinary Medicine National Taiwan University Taipei Taiwan; ^2^ Department of Basic Sciences, College of Veterinary Medicine Mississippi State University Starkville Mississippi; ^3^ Department of Animal Science and Technology National Taiwan University Taipei Taiwan; ^4^ Technical Service Center National Animal Industry Foundation Pingtung City Taiwan

**Keywords:** 4‐androstene‐3,17‐dione, liquid chromatography–tandem mass spectrometry, progesterone, shell eggs, testosterone

## Abstract

Steroid hormones are often used in animal agriculture but are currently banned for use in domesticated fowl because residual hormones could be present in eggs for human consumption. Egg samples from eight common commercial poultry layer breeds (Hy‐Line W‐36, Hy‐Line Brown, ISA‐White, ISA‐Brown, Lohnmann Ultra‐Lite, Lohnmann‐Brown, Hisex White, Hisex Brown) in Taiwan were screened for a combination of 15 natural and synthetic steroid hormones by liquid chromatography–tandem mass spectrometry (LC‐MS/MS) for consumer assurance. Only natural hormones such as progesterone, 4‐androstene‐3,17‐dione, and testosterone were detected. Regarding each breed, the interaction effect (age × shell color), main effect (age or shell color), and blocking effect (lighting system) were further analyzed by using 2 × 2 factorial arrangement of treatment in a randomized block design. We also discovered associations between yolk steroid hormone levels and laying hen age, as well as lighting conditions. Additionally, we found a correlation between hormone levels and eggshell color, suggesting a potential role in brown pigmentation. Ultimately, we concluded that detectable steroid hormone levels in eggs were not a consumer health risk. Furthermore, these data provide empirical hormone concentrations in various types of commercial layer breeds for future research.

## 1 INTRODUCTION

Steroid hormones are produced naturally in the human body and have significant impacts on several biological processes including growth, development, and reproduction (Edwards, 2005). Sex steroid hormones are typically synthesized in the gonads and are subsequently released into the bloodstream to exert their effects by binding to specific intracellular receptors. Because these molecules have critical roles in the body, they are exogenously administered for livestock production purposes to improve weight gain and feed efficiency (Andersson & Skakkebaek, 1999). However, inappropriate use of hormones such as an incorrect administration protocol or withdrawal period could result in residues in the animal products that could be passed on to consumers and pose possible health risks (Aksglaede, Juul, Leffers, Skakkebaek, & Andersson, 2006). Therefore, it is important to establish a reliable analytical method for hormone residues in foods of animal origin for the control and surveillance to reduce human exposure and ensure consumer protection.

Eggs are an animal product and major food source in the common human diet. In the early 1950s, synthetic estrogens were commercially used as a feed additive to increase the weight of poultry (Reed & Fenton, 2013). Although the use of hormones for increased mass in animals has been prohibited in the European Community since 1989, research has revealed that avian egg yolks contain substantial levels of maternally derived sex steroid hormones (Schwabl, Mock, & Gieg, 1997). Estrogen, progesterone, and testosterone have been detected in the eggs of domesticated fowl; however, the underlying mechanism of maternal hormone deposition during egg production remains unknown (Lipar, Ketterson, Nolan, & Casto, 1999). These sex hormones are responsible for the maturation of ovulatory follicles, egg formation, as well as offspring development, and are regulated by both genetic and environmental factors (Gil, 2008). Because there are passive effects of these factors upon consumer ingestion, concern is warranted for higher risk groups in which assimilation of these hormones pose as a potential health hazard (Passantino, 2012). Therefore, there is a critical need for development of a robust and sensitive analytical method for quantitative evaluation of these steroid hormones.

Currently, detection of steroid hormones in eggs has been assessed using gas chromatography–mass spectrometry (GC‐MS) methods (Hartmann, Lacorn, & Steinhart, 1998). However, GC‐MS involves a tedious derivatization process with the potential for loss of molecular information at each transfer step (Fritsche, Schmidt, & Steinhart, 1999). Furthermore, the impact of different poultry housing systems, as well as variation in eggshell pigmentation, has not been studied in relation to sex hormone levels in various commercial avian species. This study uses the procedure of liquid chromatography coupled with tandem mass spectrometry (LC‐MS/MS) without derivatization to determine the effects of housing systems on residue levels of 15 steroid hormones in white or brown pigmented eggs produced by four different strains of commercial layers (Hy‐Line W‐36, Hy‐Line Brown, ISA‐White, ISA‐Brown, Lohnmann Ultra‐Lite, Lohnmann‐Brown, Hisex White, Hisex Brown) common to the Taiwan consumer market. The aim of this study was to provide an accurate measurement of steroid hormone levels in commonly obtainable poultry eggs available for human consumption in Taiwan to provide a fundamental reference and assist nutritionists and medical professionals so that they may accurately estimate hormonal intake for patients.

## 2 MATERIALS AND METHODS

### 2.1 Egg collection

Sixty fowls for each specific strain of layer breed (Hy‐Line W‐36, Hy‐Line Brown, ISA‐White, ISA‐Brown, Lohnmann Ultra‐Lite, Lohnmann‐Brown, Hisex White, Hisex Brown) were raised either under natural or artificial lighting system conditions. Layers were exposed to either 16 hr of artificial light or 12 hr of natural light a day. A total of 15 egg samples from each layer breed strain at 30‐ or 60‐weeks of age and under both lighting conditions were randomly collected and stored at 4°C. Prior to testing, the contents of five eggs from each cohort were pooled together for subsequent analyses.

### 2.2 Reagents and standards

Analytical standards for 17α‐ethinylestradiol (EE2), 17β‐estradiol (E2β), diethylstilbestrol (DES), estrone (E1), estriol (E3), hexestrol (HEX), progesterone (PS), testosterone (TS), 4‐androstene‐3,17‐dione (AD), 17α‐methyltestosterone (MTS), testosterone propionate (TSP), as well as internal standards (IS) for β‐estradiol‐d2 (E2‐d2) and progesterone‐d9 (PS‐d9), were all obtained from Sigma Aldrich. Additionally, 17α‐estradiol (E2α) and 17α‐hydroxyprogesterone (HPS) were purchased from Dr. Ehrenstorfer. Dienestrol (DE) and α‐zearalanol (ZAL) were supplied through TCI EUROPE N.V., and Kanto Chemical Co., Inc, respectively. IS for diethylstilbestrol‐d8 (DES‐d8) and hexestrol‐d4 (HEX‐d4) were procured from Toronto Research Chemicals Inc. Bondesil C18 bulk sorbent and Bondesil PSA bulk sorbent were acquired from Agilent. HPLC‐grade acetonitrile and ammonia solvents were purchased from J. T. Baker, and Merck Millipore, respectively. Lastly, Waters Xbridge BEH C18, used to analyze negatively charged hormones, was obtained from Waters while Phenomenex Gemini C18, used to analyze positively charged hormones, was obtained from Phenomenex.

### 2.3 Preparation of standards and working solutions

Standard stock solutions at 1 mg/ml were prepared in methanol and stored at –20°C prior to use. Appropriate concentrations of working solutions were obtained based on the calibration curve range and were achieved by serial dilution of stock solutions in methanol.

### 2.4 Sample extraction and cleanup

In centrifuge tubes, 2 g of preserved homogenized egg samples were suspended in 5 ml of ddH_2_O, and each sample was subsequently spiked with 50 μl of the respective IS working solution. Afterward, 10 ml of 1% acetonitrile followed by 5 g of anhydrous sodium acetate was added to the centrifuge tubes and mixed by vortex for 2 min at high speed. The samples were then centrifuged for 10 min at 39,000 *g* at 4°C and the supernatants were transferred to new tubes. This extraction procedure was repeated once more before combing the sample supernatants. For sample cleanup, the sample supernatants were passed through Bondesil C18 cartridges under 3,000 *g* for 10 min and then placed under a nitrogen stream at 50°C until eluent evaporation. The extracts were dissolved in 1 ml acetonitrile, centrifuged at 21,000 *g* for 10 min, filtered through 0.22‐μm nylon filters, and then injected directly onto the liquid chromatography/tandem mass spectrometer (LC‐MS/MS) for immediate analysis.

### 2.5 LC‐MS/MS analysis

LC‐MS/MS analysis was carried out on an Agilent 1290 series chromatographic system and an Agilent 6460 triple quadrupole mass spectrometer (Agilent Technologies) with electrospray ionization (ESI). Separation of negatively charged hormones was achieved in a Waters Xbridge BEH C18 column (2.1 mm × 150 mm, 3.5 μm), whereas separation of positively charged hormones was achieved in a Phenomenex Gemini C18 column (2.0 mm × 100 mm, 3.0 μm). The mobile‐phase gradient was composed of solvent A (0.1% ammonia) and solvent B (acetonitrile) with a flow rate of 300 μl/min and was performed as follows: 0–0.5 min (80% A, 20% B), 1–6 min (60% A, 40% B), 6–7 min (55% A, 45% B), and 7–9 min (10% A, 90% B). The injection volume was 15 μl, and the column was set at 40°C. For mass spectrometric analysis, the ESI settings were as follows: capillary voltage 3.0 kV; desolvation gas flow rate 9 L/min at 350°C; and nebulizer pressure 45 psi. MS‐MS acquisition was performed in multiple‐reaction‐monitoring (MRM) mode. The optimized settings for product ions of each hormone are shown in Table 1.

**Table 1:** Mass spectrometry parameters for each hormone using multiple‐reaction‐monitoring (MRM) mode

Ionization	Hormone	Precursor ion (m/z)	Product ion (m/z)	Cone (V)	Collision energy (eV)
Negative	Dienestrol	265.1	93.1	144	21
	Diethylstilbestrol	267.1	237.2	124	17
	17α‐Estradiol	271.2	145.2	185	37
	17β‐Estradiol	271.1	183.2	110	33
	Estrone	269	145.2	115	29
	Estriol	287.2	171.2	110	33
	17α‐Ethinylestradiol	295.2	145	105	33
	Hexestrol	269.1	119.1	95	29
	α‐Zearalanol	321.2	277.3	110	17
	Diethylstilbestrol‐d8	275.2	259.1	140	21
	β‐Estradiol‐d2	273.2	147.1	120	37
	Hexestrol‐d4	273.2	136.1	120	9
Positive	17α‐Hydroxyprogesterone	331.2	97.1	135	29
	Progesterone	315.2	108.9	144	25
	Progesterone‐d9	324.3	100.1	100	22
	Testosterone	289.2	96.9	144	21
	4‐Androstene‐3,17‐dione	287.2	109	45	24
	17α‐Methyltestosterone	303.2	96.9	144	25
	Testosterone propionate	345.2	96.9	144	21

### 2.6 Method validation

The method was validated according to the guidelines set by the European Medicines Agency (van Amsterdam et al., 2013). The linearity of the 15 steroid standard curves was prepared with five concentration points within the range of 0–200 ng/ml. The calibration curves were constructed by weighted (1/*x*) least‐squares linear regression analysis and were described by the following linear equation: *y* = a*x* ± b. Recoveries were assessed by comparing the peak areas of spiked egg samples with those of eggs that were not spiked at three concentrations (*n* = 6). The precision was expressed using the relative standard deviation while the limits of detection and quantification were calculated as 3 × and 10 × the signal‐to‐noise ratios, respectively, in the spiked egg samples.

### 2.7 Statistical analysis

The experiment was conducted using 2 × 2 factorial arrangement of treatment in a randomized block design. Regarding each breed, the interaction effect (age × shell color, *n* = 6), main effect (age or shell color, *n* = 12), and blocking effect (lighting system, *n* = 12) were analyzed. Data were presented as mean ± *SD*. All significant differences in the interaction effect, main effect, and blocking effect were tested using an ANOVA test at a 0.05 probability level (*p* ≤ 0.05). When a significant difference in the interaction effect was determined, the least significant difference (LSD) test at a 0.05 probability level (*p* ≤ 0.05) was used to test the differences between combination treatments. All statistical analyses of data were performed using SAS Institute (Version 2002, Cary, NC, USA). Correlation between different variables was analyzed using Pearson's correlation co‐efficient in SPSS for Windows, version 20.0 (IBM‐SPSS).

## 3 RESULTS AND DISCUSSION

For several years, the presence of regulated hormone residues in food that exceeds the maximum prescribed limits has been a concern for public health. Animal studies have reported adverse effects, such as altered reproductive development, due to prenatal sex steroid hormone exposure (Seegers, van Aswegen, Nieuwoudt, & Joubert, 1991). Additionally, hormones derived from animal products have endocrine‐disrupting effects in humans (Nakajima et al., 2015); therefore, steroid hormones may have a function in multiple diseases, including cancer (Krieger, 2008; Nachman & Smith, 2015). Furthermore, food containing high levels of sex steroid hormones may pose a problem for professional athletes if ingestion results in the detection of hormones on the banned‐substance list, which could prompt athlete disqualification (Thevis, Kuuranne, Geyer, & Schänzer, 2013). Consequently, it is extremely important for consumer protection to implement a standard monitoring system for food hormone residue levels. This is the first study to determine the concentration of sex steroid hormones in eggs from eight common commercial layer breed strains under different housing conditions, which holds great significance because eggs are a common staple in both Western and Eastern diets.

Although the use of steroid hormones in poultry is completely illegal in Taiwan, some commonly used synthetic hormones were included in our analyses to ensure the absence of any form of the molecules. Table 2 showed the linearity and recovery of the 15 natural and synthetic sex hormones. The calibration curves for the steroid hormones exhibited good linearity with correlation co‐efficient (*r* ^2^) values of more than 0.99, which met the criteria recommended by European legislation (Commission of the European Communities, 2002). Moreover, the recovery rate was within the range of 72.9% and 105.6%, which was in accordance with the Codex Alimentarius established values of 70%–120% (Alimentarius, 2017).

**Table 2:** Linearity and recovery of 15 steroid hormones

Compounds	Produced naturally or synthetically	Retention time (min)	Slope	Intercept	*r* ^2^	Linear range (ng/ml)	% Recovery
α‐zearalanol	Natural	2.242	3.0131	1.4474	0.9983	10–200	101.6–105.6
Estriol	Natural	2.651	0.8629	0.0091	0.9962	2–40	83.9–88.3
17α‐ethinylestradiol	Synthetic	5.659	0.7513	0.0026	0.9983	4–80	82.1–85.3
α‐Estradiol	Natural	5.372	1.9640	–0.0057	0.9984	2–40	84.2–84.9
β‐Estradiol	Natural	4.755	1.0933	0.0099	0.9984	2–40	79.7–82.5
Dienestrol	Synthetic	6.120	2.5528	–0.0110	0.9982	2–40	86.0–88.0
Diethylstilbestrol	Synthetic	5.900	0.9935	–0.0049	0.9991	2–40	93.0–98.4
Estrone	Natural	5.914	4.4708	0.0289	0.9977	2–40	74.5–78.0
Hexestrol	Synthetic	7.162	2.4410	0.0003	0.9993	2–40	98.3–100.8
17α‐Hydroxyprogesterone	Natural	6.129	0.7642	–0.0093	0.9985	2–200	98.3–104.9
17α‐Methyltestosterone	Synthetic	5.864	1.1204	–0.0159	0.9985	2–200	90.2–97.6
4‐androstene‐3,17‐dione	Natural	5.948	1.0695	–0.0150	0.9986	2–200	94.6–106.2
Testosterone	Natural	5.314	1.3261	–0.0190	0.9985	2–200	100.1–102.2
Progesterone	Natural	8.017	1.4083	–0.0199	0.9985	2–200	83.1–91.6
Testosterone propionate	Synthetic	10.336	1.1796	–0.0234	0.9966	2–200	72.9–78.7

A previous study using LC‐MS/MS detected quantitative amounts of PS, TS, and AD residues in eggs purchased from supermarkets (Mi et al., 2014). Similarly, we found only naturally occurring steroid hormones in egg samples, indicating that no additional or synthetic steroid hormones are being administered to commercial layers in Taiwan. All the egg samples contained fairly high amounts of natural PS (Geometric Mean Range: 53.58–167.50 ppb; Table 3), which is known to influence bone growth during bird development (Schär, 1967). Significantly higher levels of PS were observed in eggs from layers at the age of 60 weeks compared to 30 weeks, particularly those laid by the Hy‐Line, ISA, and Hisex breeds (*p ≤ *0.05, *t* test; Table 3). Although the production of PS varies in different layer breeds, our data align with the results of an earlier study (Liu, Long, & Bacon, 2002); in which a higher concentration of PS and a lower concentration of luteinizing hormone were associated with a decline in egg production with reproductive period progression in turkeys. Therefore, it is possible that elevated circulating PS that occurs with age in layer breeds could be deposited in yolks during egg production and development.

**Table 3:** Effects of hen age, shell color, and housing systems on steroid hormones in eggs produced by different layer breeds

Breed	Steroid hormone	Age		Shell color		House system		Age	Shell color	Age × shell color	House system
		30	60	Brown	White	Artificial light	Natural light	*p*‐value			
Hy‐Line	AD (ppb)	11.67 ± 6.84	12.17 ± 4.73	16.33 ± 4.29*	7.50 ± 2.88	9.25 ± 5.01	14.58 ± 5.35*	0.61	<0.0001	0.14	<0.0001
	PS (ppb)	87.58 ± 31.54	116.50 ± 33.72*	124.00 ± 35.41*	80.08 ± 4.85	117.25 ± 36.47*	86.83 ± 27.46	0.0008	<0.0001	0.34	0.005
ISA	AD (ppb)	10.33 ± 3.87	12.25 ± 4.73	14.08 ± 4.29*	8.50 ± 1.98	11.17 ± 3.24	11.42 ± 5.37	0.15	0.0003	0.06	0.85
	PS (ppb)	92.25 ± 69.30	128.83 ± 67.46*	167.50 ± 51.12*	53.58 ± 19.77	99.58 ± 48.83	121.50 ± 86.23	0.02	<0.0001	0.65	0.13
Lohnmann	AD (ppb)	9.58 ± 3.00	13.33 ± 8.96	10.67 ± 2.31	12.25 ± 9.50	7.67 ± 2.81	15.25 ± 7.59*	0.10	0.47	0.07	0.00
	PS (ppb)	82.75 ± 35.78	107.50 ± 36.04	90.92 ± 4.87	99.33 ± 33.49	104.92 ± 27.20	85.35 ± 44.32	0.11	0.58	0.50	0.20
Hisex	AD (ppb)	9.58 ± 4.32	11.00 ± 5.53	14.08 ± 4.12*	6.50 ± 1.38	10.92 ± 5.40	9.67 ± 4.50	0.27	<0.0001	0.40	0.33
	PS (ppb)	91.08 ± 31.41	112.17 ± 52.37	136.00 ± 37.74	67.25 ± 15.54	100.83 ± 49.44	102.42 ± 42.21	0.04	<0.0001	0.0047	0.81

Note

Data are given as Mean ± *SD* (*n* = 12; three replicates for each strain). Mean values in the main effect (age or shell color) and blocking effect (housing system) with * indicate a significant difference (*p ≤ *0.05) for the same steroid hormone level within the same breed.

Abbreviations: AD, 4‐androstene‐3,17‐dione; PS, Progesterone.

In addition to the presence of PS, the eggs also exhibited substantial amounts of AD (Geometric Mean Range: 7.50–16.33 ppb; Table 3), which can be further converted to testosterone or estrone by steroidogenic enzymes for chick embryo growth progression (Benowitz‐Fredericks & Hodge, 2013; Eising, Eikenaar, Schwabl, & Groothuis, 2001). We believe that our study is the first to report the effects of lighting systems on the AD concentrations present in eggs. There were no significant differences in egg AD levels in relation to natural or artificial lighting conditions among the ISA and Hisex layer breeds (Table 3). However, the Hy‐Line and Lohnmann breeds housed under natural lighting conditions had significantly higher egg yolk AD concentrations compared to those housed in artificial lighting conditions (*p ≤ *0.05, *t* test; Table 3). Our findings are consistent with observations made by Schwabl (1996), who reported significantly higher androgen concentrations in egg collections following a 12 hr of light:12 hr of dark photoperiod compared to a 16 hr of light:8 hr of dark photoperiod. These data indicate a connection between environmental factors that affect the biological circadian rhythms of avian species and AD levels present in eggs. Moreover, significant differences in PS and AD levels in eggs from different strains of the same breed were shown to be conditional to eggshell color (*p ≤ *0.05, *t* test; Table 3). Apart from the Lohnmann breed, a remarkable increase in the PS and AD concentrations that coincided with a brown eggshell pigmentation was observed in the Hy‐Line, ISA, and Hisex layer breeds (*p* ≤ 0.05, *t* test; Table 3). A recent study on individual broilers of the same breed and under common industrial standard management reported a large variation in growth hormone levels (Wang et al., 2013), which could explain the observed differences in PS and AD levels among the layer breed strains in this study. Other than PS and AD, a small amount of TS was found in Lohnmann eggs, but not in the eggs of the other breeds (data not shown). To date, a comparison of steroid hormone concentrations in eggs from different layer breed strains has not been conducted. Aside from the extrinsic factor of lighting conditions, the results of this study showed variance in steroid hormone levels among specific layer breed strains, which could possibly be the effect of genetic or dietary factors. It is not surprising that eggs are a considerable source of hormonally active steroids and their precursors, because eggs are produced from the major female steroidogenic organs, which are responsible for steroid hormone production to maintain normal physiological embryo development (Hartmann et al., 1998). As sex hormones also have essential roles in appetite regulation, eating behavior, and energy metabolism, these findings can also provide fundamental data for the consideration of nutritionists (Hirschberg, 2012).

**Figure 1:**
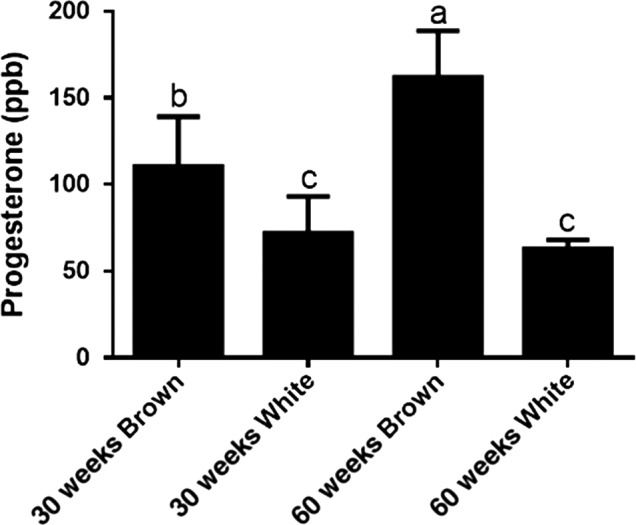
Relative progesterone levels in relation to age and eggshell color in egg yolks of the Hisex breed. Data bars without the common letter indicate a significant difference between groups (*p ≤ *0.05)

All the measured parameters in this study were evaluated for further correlation. In particular, the eggs from 60‐week‐old Hisex breed hens had significantly higher PS levels than the other cohorts (*p ≤ *0.05; Figure 1). Moreover, associations were also found between specific hormone concentrations, photoperiod, age of the layer hen, and eggshell color (Table 4). These observed correlations between the yolk steroid hormone levels can be explained by the fact that pathways for the synthesis and metabolism of different steroid hormones are connected during steroidogenesis (Lee, Volentine, & Bahr, 1998). We also observed a significant positive association between the photoperiod and yolk TS and AD concentrations among the layer breed strains. As previously mentioned, these data coordinate with a previous study which demonstrated that female canaries produce eggs with greater amounts of yolk TS when exposed to shorter lengths of daylight (Schwabl, 1996). Furthermore, as androgens are known to affect avian embryo growth, this may be a mechanism attributable to environmental influence and used to alter offspring phenotype across seasons (Lipar & Ketterson, 2000).

**Table 4:** Pearson correlations between photoperiod, breed, age, eggshell color, and hormones

Parameters	Testosterone	4‐androstene‐3,17‐dione	Progesterone
4‐androstene‐3,17‐dione	0.278*		
Progesterone	0**.**058	0.615*	
Photoperiod	0.209*	0.274*	–0.065
Breed	0.093	–0.097	–0.033
Age	0.104	0.174	0.287*
Eggshell color	–0.104	0.469*	0.567*

* *p ≤ *0.05; Pearson correlation co‐efficients (*n* = 96).

Although there was no direct association found between different layer breeds and hormone levels, significant positive correlations were observed between eggshell color and yolk AD and PS concentrations. To the authors’ knowledge, this is the first study to report a difference in egg yolk AD levels based on either a brown or white eggshell color from the same layer breeds. Although the location of brown pigment synthesis in poultry eggs has not been confirmed, certain hormonally active molecules such as PS, prostaglandins, and estradiol‐17β have been reported to enhance pigment production (Soh & Koga, 1999). Thus, continued research to reveal the underlying mechanisms for eggshell pigmentation and the involvement of hormones is necessary and remains an interesting subject for future study.

Finally, we included a table (Table 5) that presents the acceptable daily intake (ADI) recommendations of PS, TS, and 4‐androstene‐3,17‐dione established by the Joint FAO/WHO Expert Committee on Food Additives (JECFA, 1999) and the US Food and Drug Administration (FDA, 2006). Although the recommendations for AD have not yet been established, it is clearly shown that the permitted increase exposure (PIE) for the 4‐androstene‐3,17‐dione precursor hormone established by the US FDA is well below the ADI. Furthermore, the steroid hormone levels acquired during this study were far below the recommended values set by the JECFA and FDA, suggesting that there is no consumer health risk for steroid hormone residue exposure from commercial eggs produced by common layer breeds in Taiwan.

**Table 5:** Maximum daily intake recommendations by Joint FAO/WHO Expert Committee on Food Additives (JECFA) and the US Food and Drug Administration (FDA) for progesterone, 4‐androstene‐3,17‐dione, and testosterone

Criteria	Parameter	Progesterone	4‐androstene‐3,17‐dione	Testosterone
JECFA	ADI	0–0.03 mg/kg bw	–	0–0.002 mg/kg bw
	LOEL	3.3 mg/kg bw/day	–	1.7 mg/kg bw/day
FDA	PIE	1.5 μg	–	0.32 μg

Abbreviations: ADI, acceptable daily intake; LOEL, lowest‐observed‐effect level; PIE, permitted increase exposure.

## 4 CONCLUSION

The data presented in this study could add new reference values for multiple parameters for various types of commercial layers and could be useful to nutritionists as reference ranges to estimate human intake levels. Although these detectable steroid hormone levels do not pose a consumer health hazard, significant positive correlations were observed between yolk steroid hormones. Besides androgens, which are affected by the length of the photoperiod, the age of layer hens can also affect the concentrations of yolk steroid hormones. Nevertheless, further research is needed to elucidate the mechanisms to control brown pigment synthesis in the eggshell gland and determine which hormones are responsible for the development of the eggshell pigment.

## CONFLICT OF INTEREST

The authors declare that there are no conflicts of interest.

## ETHICAL STATEMENT

This report does not conduct any human or animal tests.

## References

[fsn31074-bib-0001] Aksglaede, L. , Juul, A. , Leffers, H. , Skakkebaek, N. E. , & Andersson, A. M. (2006). The sensitivity of the child to sex steroids: Possible impact of exogenous estrogens. Human Reproduction Update, 12, 341–349. 10.1093/humupd/dml018 16672247

[fsn31074-bib-0002] Alimentarius, C. (2017). Guidelines on performance criteria for methods of analysis for the determination of pesticide residues in food and feed, Vol. CXG 90.

[fsn31074-bib-0003] Andersson, A. M. , & Skakkebaek, N. E. (1999). Exposure to exogenous estrogens in food: Possible impact on human development and health. European Journal of Endocrinology, 140, 477–485. 10.1530/eje.0.1400477 10366402

[fsn31074-bib-0004] Benowitz‐Fredericks, Z. M. , & Hodge, M. (2013). Yolk androstenedione in domestic chicks (*Gallus gallus domesticus*): Uptake and sex‐dependent alteration of growth and behavior. General and Comparative Endocrinology, 193, 48–55. 10.1016/j.ygcen.2013.07.005 23871777

[fsn31074-bib-0005] Commission of the European Communities . (2002). European Commission Decision of 12 August 2002 implementing Council Directive 96/23/EC concerning the performance of analytical methods and the interpretation of results (2002/657/EC). Official Journal, L 221, 8–36.

[fsn31074-bib-0006] Edwards, D. P. (2005). Regulation of signal transduction pathways by estrogen and progesterone. Annual Review of Physiology, 67, 335–376. 10.1146/annurev.physiol.67.040403.120151 15709962

[fsn31074-bib-0007] Eising, C. M. , Eikenaar, C. , Schwabl, H. , & Groothuis, T. G. G. (2001). Maternal androgens in black‐headed gull (*Larus ridibundus*) eggs: Consequences for chick development. Proceedings of the Royal Society B: Biological Sciences, 268, 839–846. 10.1098/rspb.2001.1594 PMC1088678 11345330

[fsn31074-bib-0008] FDA . (2006). Guidance for industry: General principles for evaluating the safety of compounds used in food‐producing animals (pp. 2319–42). Washington, DC: US. Food and Drug Administration Center for Veterinary Medicines.

[fsn31074-bib-0009] Fritsche, S. , Schmidt, G. , & Steinhart, H. (1999). Gas chromatographic‐mass spectrometric determination of natural profiles of androgens, progestogens, and glucocorticoids in muscle tissue of male cattle. European Food Research and Technology, 209, 393–399. 10.1007/s002170050515

[fsn31074-bib-0010] Gil, D. (2008). Chapter 7 Hormones in avian eggs: Physiology, ecology and behavior In Brockmann H. J., Roper T. J., Naguib M., Wynne-Edwards K. E., Barnard C., Mitani J. Ed, Advances in the study of behavior 38, (pp. 337–398). Amsterdam, Netherlands: Elsevier Science 10.1016/S0065-3454(08)00007-7

[fsn31074-bib-0011] Hartmann, S. , Lacorn, M. , & Steinhart, H. (1998). Natural occurrence of steroid hormones in food. Food Chemistry, 62, 7–20. 10.1016/S0308-8146(97)00150-7

[fsn31074-bib-0012] Hirschberg, A. L. (2012). Sex hormones, appetite and eating behaviour in women. Maturitas, 71, 248–256. 10.1016/j.maturitas.2011.12.016 22281161

[fsn31074-bib-0013] JECFA (Joint FAO/WHO Expert Committee on Food Additives) . (1999). Evaluation of certain veterinary drugs residues in food. Fifty‐second report of the joint FAO/WHO expert committee on food additives. World Health Organization Technical Report Series, 893, 2319–102. World Health Organisation, Geneva.

[fsn31074-bib-0014] Krieger, N. (2008). Hormone therapy and the rise and perhaps fall of US breast cancer incidence rates: Critical reflections. International Journal of Epidemiology, 37, 627–637. 10.1093/ije/dyn055 18375445

[fsn31074-bib-0015] Lee, K. A. , Volentine, K. K. , & Bahr, J. M. (1998). Two steroidogenic pathways present in the chicken ovary: Theca layer prefers delta 5 pathway and granulosa layer prefers delta 4 pathway. Domestic Animal Endocrinology, 15, 2319–2326. 10.1016/S0739-7240(97)00057-X 9437580

[fsn31074-bib-0016] Lipar, J. L. , & Ketterson, E. D. (2000). Maternally derived yolk testosterone enhances the development of the hatching muscle in the red‐winged blackbird *Agelaius phoeniceus* . Proceedings of the Royal Society B: Biological Sciences, 267, 2005–2010. 10.1098/rspb.2000.1242 PMC1690769 11075714

[fsn31074-bib-0017] Lipar, J. L. , Ketterson, E. D. , Nolan, V. Jr , & Casto, J. M. (1999). Egg yolk layers vary in the concentration of steroid hormones in two avian species. General and Comparative Endocrinology, 115, 220–227. 10.1006/gcen.1999.7296 10417235

[fsn31074-bib-0018] Liu, H. K. , Long, D. W. , & Bacon, W. L. (2002). Interval between preovulatory surges of luteinizing hormone increases late in the reproductive period in Turkey hens. Biology of Reproduction, 66, 1068–1075. 10.1095/biolreprod66.4.1068 11906927

[fsn31074-bib-0019] Mi, X. , Li, S. , Li, Y. , Wang, K. , Zhu, D. , & Chen, G. (2014). Quantitative determination of 26 steroids in eggs from various species using liquid chromatography‐triple quadrupole‐mass spectrometry. Journal of Chromatography A, 1356, 54–63. 10.1016/j.chroma.2014.05.084 25017396

[fsn31074-bib-0020] Nachman, K. E. , & Smith, T. J. (2015). Hormone use in food animal production: Assessing potential dietary exposures and breast cancer risk. Current Environmental Health Reports, 2, 2319–14. 10.1007/s40572-014-0042-8 26231238

[fsn31074-bib-0021] Nakajima, T. , Tsuruoka, Y. , Kanda, M. , Hayashi, H. , Hashimoto, T. , Matsushima, Y. , … Takano, I. (2015). Determination and surveillance of hydrocortisone and progesterone in livestock products by liquid chromatography‐tandem mass spectrometry. Food Additives & Contaminants. Part A, Chemistry, Analysis, Control, Exposure & Risk Assessment, 32, 1833–1841. 10.1080/19440049.2015.1084053 26284419

[fsn31074-bib-0022] Passantino, A. (2012). A bird's eye view of veterinary medicine InPerez‐Marin C. C. (Ed.), Steroid hormones in food producing animals (1st edn, pp. 33–50). Croatia: InTech.

[fsn31074-bib-0023] Reed, C. E. , & Fenton, S. E. (2013). Exposure to diethylstilbestrol during sensitive life stages: A legacy of heritable health effects. Birth Defects Research, Part C: Embryo Today‐Reviews, 99, 134–146. 10.1002/bdrc.21035 PMC3817964 23897597

[fsn31074-bib-0024] Schär, B. (1967). Die Wirkung von Progesteron und Progesteronmetaboliten auf das Wachstum von embryonalem Knorpel in vitro. Experientia, 23, 716–717. 10.1007/BF02154131 6062881

[fsn31074-bib-0025] Schwabl, H. (1996). Environment modifies the testosterone levels of a female bird and its eggs. Journal of Experimental Zoology, 276, 157–163. 10.1002/(SICI)1097-010X(19961001)276:2<157:AID-JEZ9>3.0.CO;2-N 8900078

[fsn31074-bib-0026] Schwabl, H. , Mock, D. W. , & Gieg, J. A. (1997). A hormonal mechanism for parental favouritism. Nature, 386, 231 10.1038/386231a0 9069278

[fsn31074-bib-0027] Seegers, J. C. , van Aswegen, C. H. , Nieuwoudt, B. L. , & Joubert, W. S. (1991). Morphological effects of the catechol estrogens on cells of the seminiferous tubules of Sprague‐Dawley rats. Andrologia, 23, 339–345. 10.1111/j.1439-0272.1991.tb02576.x 1666271

[fsn31074-bib-0028] Soh, T. , & Koga, O. (1999). Effects of phosphate, prostaglandins, arachidonic acid and arginine vasotocin on oviposition and pigment secretion from the shell gland in Japanese quail. British Poultry Science, 40, 131–134. 10.1080/00071669987962 10405049

[fsn31074-bib-0029] Thevis, M. , Kuuranne, T. , Geyer, H. , & Schänzer, W. (2013). Annual banned‐substance review: Analytical approaches in human sports drug testing. Drug Testing and Analysis, 5, 2319–19. 10.1002/dta.1441 23229872

[fsn31074-bib-0030] van Amsterdam, P. , Companjen, A. , Brudny‐Kloeppel, M. , Golob, M. , Luedtke, S. , & Timmerman, P. (2013). The European bioanalysis forum community's evaluation, interpretation and implementation of the European Medicines Agency guideline on bioanalytical method validation. Bioanalysis, 5, 645–659. 10.4155/bio.13.19 23484783

[fsn31074-bib-0031] Wang, J. , Wu, S. G. , Zhang, H. J. , Yue, H. Y. , Xu, L. , Ji, F. , … Qi, G. H. (2013). Trimethylamine deposition in the egg yolk from laying hens with different FMO3 genotypes. Poultry Science, 92, 746–752. 10.3382/ps.2012-02313 23436525

